# Effect of temperature on life table parameters of the predatory soil mite *Blattisocius mali* (Acari: Blattisociidae) fed on *Tyrophagus putrescentiae* (Acari: Acaridae)

**DOI:** 10.1038/s41598-026-66347-x

**Published:** 2026-08-10

**Authors:** Manoj Kumar Jena, Katarzyna Michalska, Marcin Studnicki

**Affiliations:** 1 Department of Plant Protection, Institute of Horticultural Sciences, Warsaw University of Life Sciences, Nowoursynowska 159, Warsaw, 02-776 Poland; 2 Department of Biometry, Institute of Agriculture, Warsaw University of Life Sciences, Nowoursynowska 159, Warsaw, 02-776 Poland

**Keywords:** Biological parameters, Mesostigmata, Mould mite, Population parameters, Soil mite, Ecology, Ecology, Zoology

## Abstract

Temperature impacts the development, survival, and fecundity of edaphic predatory mites. This study examined the biological and life table parameters of the edaphic predatory mite *Blattisocius mali* (Oudemans) (Acari: Blattisociidae) feeding on *Tyrophagus putrescentiae* (Schrank) (Acari: Acaridae) at four different temperatures, 15 °C, 20 °C, 25 °C, and 30 °C. The developmental period, reproductive period, and adult longevity of *B. mali* shortened with rising temperature. The lower temperature threshold and thermal constant ranged between 13.02 and 13.73 °C, and 83.36–89.04 ^o^D, respectively. Mated females had longer ovipositional periods and longevity, and greater fecundity and egg-hatching percentage than those of virgin females. The age-specific fecundity and survival rate were satisfactorily described by temperature-dependent models. The peak fecundity rate of both mated (2.12 eggs/female/day) and virgin females (1.92 eggs/female/day) was obtained at 25 °C. The sex ratio of mated females was female-biased and did not vary with temperature. *Blattisocius mali* had significantly higher values of finite rate of increase and intrinsic rate of increase at 25 °C and 30 °C, while it had higher values of net reproductive rate and gross reproductive rate at 20 °C and 25 °C. The mean generation time and doubling time were the longest at 15 °C. This study demonstrated that *B. mali* could develop and reproduce at temperatures ranging from 15 °C to 30 °C, and the prey *T. putrescentiae* have approximately similar temperature thresholds for development, positioning it as a viable option for the biological control of acarid mite pests within this temperature range.

## Introduction

The global temperature began to rise after the Industrial Revolution due to climate change, and the global mean temperature is expected to reach 20 °C around 2180^1^. This rise in global temperature has significant implications for soil temperature and moisture levels^2^, interfering with the functioning of edaphic predatory mites used in biological control and subsequently affecting the future of sustainable agriculture. Temperature can affect species diversity and richness, and the abundance of soil mite communities^3^. It is an important component of predator-prey interactions, influencing soil mites’ metabolic rate and feeding activity^4,5^. Additionally, temperature can affect population dynamics, development, survival, reproduction, activity, and life table parameters of edaphic predatory mites^6–8^.

Life tables of insect and mite predators and pests are important for comprehensively understanding their population ecology^9^. There are two primary categories of life tables: individually-reared and group-reared life table^9^. Life tables form a fundamental basis of successful mass-rearing when used to assess the impact of population-level effects. They are also important to the success of biological control, sterile insect technique (SIT), male annihilation technique (MAT), and integrated pest management (IPM) programs^9–11^. These are used to determine the optimal interval between releases to avoid predation gaps and maintain continuity in the predatory capacity of the natural enemies^12^. Integrating life table data with predation rate facilitates the forecasting of pest population dynamics in response to the influence of one or more predators^13^. A comprehensive understanding of the impact of temperature on a natural enemy’s biological and life table parameters is crucial for its evaluation as a biocontrol agent in different geographical locations^14^. In mite predators, the biological and life table parameters can be affected by various factors, such as temperature^6–8^, humidity^15,16^, photoperiod^17^, and prey type^18–22^, which are usually studied to understand their effects on the population dynamics of the predator-prey system.

The mites belonging to the family Blattisociidae are free-living predators that show promise as biocontrol agents against a variety of arthropod pests. These mites are frequently recorded in storage, on plants, or in association with other mites, insects, rodents, birds, and fungi^23^. Among blattisociid mites, *Blattisocius tarsalis* (Berlese) (Acari: Blattisociidae), *B. keegani* (Fox), *B. dentriticus* (Berlese), and *B. mali* (Oudemans) are more extensively studied. *Blattisocius mali* is a generalist predator and has been reported to prey on a wide variety of arthropod pests, such as, the mould mite *Tyrophagus putrescentiae* (Schrank) (Acari: Acaridae), bulb mite *Rhizoglyphus robini* (Claparède) (Acari: Acaridae), two-spotted spider mite *Tetranychus urticae* (Koch) (Acari: Tetranychidae), western flower thrips, *Frankliniella occidentalis* (Pergande) (Thysanoptera: Thripidae), drosophilid flies *Drosophila melanogaster* (Meigen) and *D. hydei* (Sturtevant) (Diptera: Drosophilidae), potato tuber moth *Phthorimaea operculella* (Zeller) (Lepidoptera: Pyralidae), and fungus gnat, *Lycoriella auripila* (Winnertz) (Diptera: Sciaridae)^18,22–28^. In addition to arthropod pests, it can feed on nematodes, such as the rhabditid nematode *Rhabditis sccanica* (Allgén)^18^. Recent studies by Jena et al.^29,30^ and Michalska et al.^31^ showed that *B. mali* showed strong potential in reducing the population of *T. putrescentiae*, a globally prevalent pest that poses a serious threat to stored products^8,32^, stored grains^33^, mushroom farms^34^, and horticultural crops^35^. This mite can develop and reproduce in a wide range of temperatures, ranging from 10.4 °C to 34.8°C^36^ and a humidity level of more than 70%^37^.

So far, the biological and life table parameters of *B. mali* have been evaluated at 25°C^18–22^. The effect of temperature on the developmental and life table parameters of blattisociid mites *B. tarsalis* and *Lasioseius* sp. has been studied^8,38–40^. Although our previous study^31^ shows that *B. mali* is highly efficient in reducing mould mite eggs across a wide range of temperatures, there is no report on the impact of temperature on the biological and life table parameters of this predatory mite. To the best of our knowledge, this study represents the first report of the effect of temperature on the biological and life table parameters of *B. mali*. This study aimed to investigate the biological and life table parameters of *B. mali* at different temperatures, 15 °C, 20 °C, 25 °C, and 30 °C, using *T. putrescentiae* eggs and larvae as prey, to evaluate its thermal fitness. It forms a fundamental basis for determining the optimal temperature for successful mass rearing of *B. mali* and plays an important role in the success of biological control and integrated pest management programs of acarid mite pests in different geographical regions. As temperature considerably impacts the development, survival, reproduction, activity, and life table parameters of blattisociid and other edaphic mites^6–8,38^, we expected that temperature might influence the biological and life table parameters of *B. mali.*

## Results

The results of Generalized Linear Model (GLM) and two-way analysis of Variance (ANOVA) showed that both temperature and the type of individual (male, female, mated female, or virgin female) significantly affected the larval period, life cycle, adult pre-ovipositional period (APOP), ovipositional period, fecundity, egg hatching percentage, and adult longevity of *B. mali* (Table 1). While temperature significantly affected the egg, protonymphal, deutonymphal, and total pre-ovipositional period (TPOP) of *B. mali*, the type of individual did not show a significant effect on these parameters (Table 1). Additionally, the interaction between temperature and the type of individual was significant for the larval period, egg-hatching percentage, and adult longevity, and it was nonsignificant for all other biological parameters (Table 1).

*Blattisocius mali* females and males completed development from egg to adult emergence at all tested temperatures (Table 2). Notably, males developed more quickly than females at all tested temperatures. The egg period of both females (*F* = 16.295, *P* < 0.0001) and males (*F* = 16.295, *P* < 0.0001) varied significantly at different temperatures and shortened substantially when exposed to a temperature of 15 °C to 30 °C, without showing any significant differences between females and males (*P* > 0.05) at all tested temperatures. The larval period of females (*F* = 13.22, *P* < 0.0001) shortened with a rise in temperature from 15 °C to 25 °C and then remained consistent up to 30 °C. The larval period of males was significantly longer at 15 °C than at other tested temperatures (*P* < 0.01), but it did not differ significantly at 20 °C, 25 °C, and 30 °C (*P* > 0.05). At 15 °C and 20 °C, the larval period of females was significantly longer than that of males (*P* < 0.05), whereas no significant difference was observed between 25 °C and 30 °C (*P* > 0.05). Further, the protonymphal period, deutonymphal period, and life cycle of both females (*F* = 17.345, *P* < 0.0001; *F* = 21.559, *P* < 0.0001; *F* = 11.2, *P* < 0.0001) and males (*F* = 6.822, *P* < 0.0001; *F* = 7.723, *P* < 0.0001; *F* = 5.40, *P* < 0.0001) were significantly longer at 15 °C and shortened with rising temperature up to 30 °C (*P* < 0.05). There was no significant difference in the protonymphal period of females and males (*P* > 0.05) at all tested temperatures. However, at 30 °C, the deutonymphal period of females was significantly longer than that of males (*P* < 0.05), without showing any significant difference at other tested temperatures (*P* > 0.05). Additionally, the life cycle of females was significantly longer than that of males at 20 °C and 30 °C (*P* < 0.05), whereas there was no significant difference in the life cycle of females and males at 15 °C and 25 °C (*P* > 0.05).

Based on these results, the lower temperature threshold and thermal constant (*k*)^41^ for the development of each life stage of *B. mali* were estimated by a linear model (Table 3). The estimated lower temperature thresholds for the development of life stages of *B. mali* females and males were the lowest (4.0 °C and 3.4 °C, respectively) for larvae and the highest (13.6 °C and 12.11 °C, respectively) for deutonymph, with mean values of 13.7 °C and 13.0 °C, respectively. Thermal constants of 89.0 ^o^D and 83.4 ^o^D were required for the complete development of immature life stages of *B. mali* females and males, respectively (Table 3).

Both mated and virgin females of *B. mali* reproduced successfully and laid eggs at all tested temperatures (Table 2). The APOP of both mated (*F* = 13.28, *P* < 0.0001) and virgin (*F* = 62.26, *P* < 0.0001) females varied significantly at different temperatures. For mated females, the shortest APOP was observed at 25 °C and 30 °C, while virgin females showed the shortest APOP at 25 °C (Table 2). Notably, at 30 °C, virgin females had a significantly longer APOP compared to mated females, although no significant differences were noted at other tested temperatures (*P* > 0.05). The TPOP of both mated (*F* = 6.17, *P* < 0.0001) and virgin (*F* = 27.80, *P* < 0.0001) females varied significantly at different temperatures and was the longest at 15 °C, and it did not differ significantly between mated and virgin females at all tested temperatures. The ovipositional period of both mated (*F* = 283.19, *P* < 0.0001) and virgin females (*F* = 415.87, *P* < 0.0001) was significantly affected by temperature and was the longest at 15 °C and shortened with rising temperature up to 30 °C. The ovipositional period of mated females was significantly longer than that of virgin females at 15 °C, 25 °C, and 30 °C (*P* < 0.05), and it did not significantly differ at 20 °C (*P* > 0.05). The postovipositional period was reported only in virgin females at 30 °C, while it was not observed at other tested temperatures. Fecundity of both mated (*F* = 316.38, *P* < 0.0001) and virgin females (*F* = 292.41, *P* < 0.0001) was significantly higher at 20 °C and 25 °C as compared to other tested temperatures. The fecundity of mated females was significantly higher than that of virgin females at all tested temperatures. The egg hatching percentage of mated (*F* = 33.38, *P* < 0.0001) and virgin females (*F* = 106.46, *P* < 0.0001) was the highest at 25 °C and decreased with decreasing temperature up to 15 °C. The egg-hatching percentage of mated females was significantly higher than that of virgin females at all tested temperatures (*P* < 0.05). Virgin females of *B. mali* gave rise to only male progeny (100%), while mated females produced progeny of both sexes (Haplodiploidy). The sex ratio of mated females was female-biased and did not differ significantly at all tested temperatures (*P* > 0.05). Adult longevity of mated (*F* = 275.35, *P* < 0.0001) and virgin females (*F* = 363.78, *P* < 0.0001), and males (*F* = 230.78, *P* < 0.0001) was the longest at 15 °C and shortened with rising temperature. Adult longevity of mated females was significantly longer than that of virgin females at 15 °C, 25 °C, and 30 °C, but it did not differ significantly at 20 °C (*P* > 0.05). Adult longevity of males was significantly shorter than that of virgin females at 15 °C, 20 °C, and 30 °C, but it did not differ significantly at 25 °C (*P* > 0.05).

**Fig. 1:**
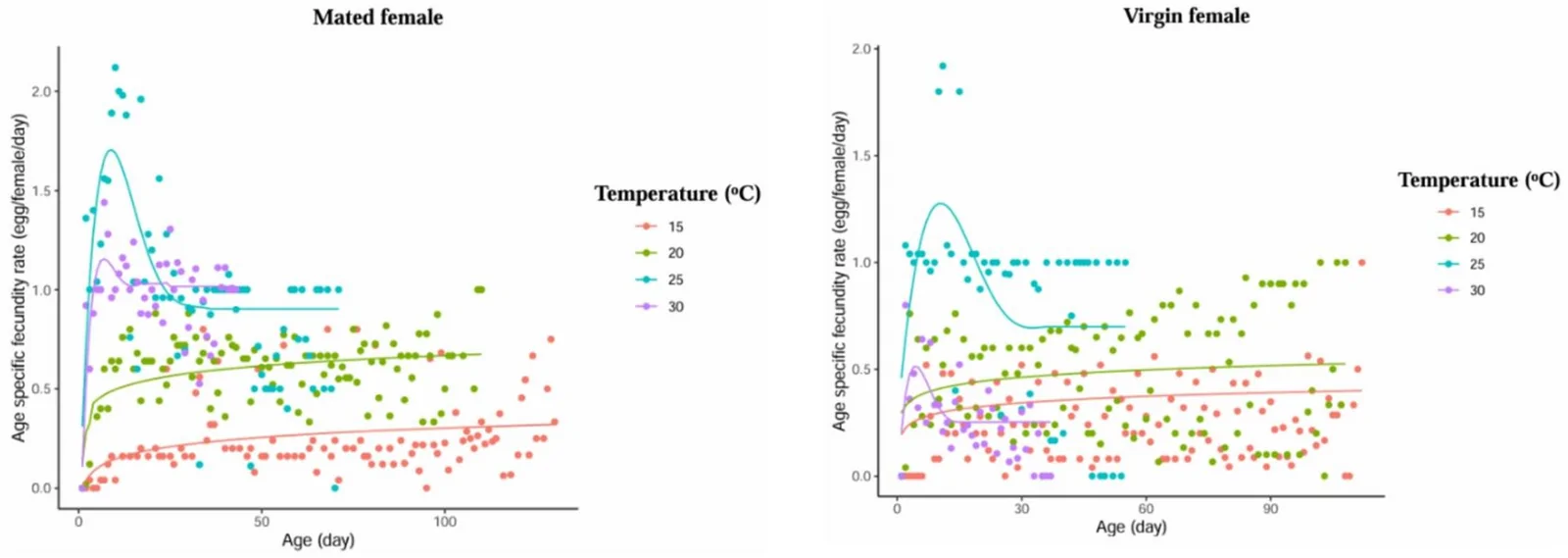
The age-specific fecundity rate (observed data: *solid dots* and Maxima function: *solid line*) of *Blattisocius mali* mated and virgin females at four different temperatures.

Daily age-specific fecundity rate, obtained by the Maxima function for both mated and virgin females at different temperatures, is given in Fig. 1. The Maxima function satisfactorily described the experimental data for both mated (*R*^*2*^ = 0.972–0.982) and virgin females (*R*^*2*^ = 0.972–0.985) and predicted the day on which the maximum fecundity rate occurred (Table 4). The maximum fecundity rate predicted by this function at 10 °C, 15 °C, 20 °C, and 25 °C were at 87, 89, 11, and 24 days, respectively, for mated females, while these were at 83, 76, 10, and 21 days for virgin females.

**Fig. 2:**
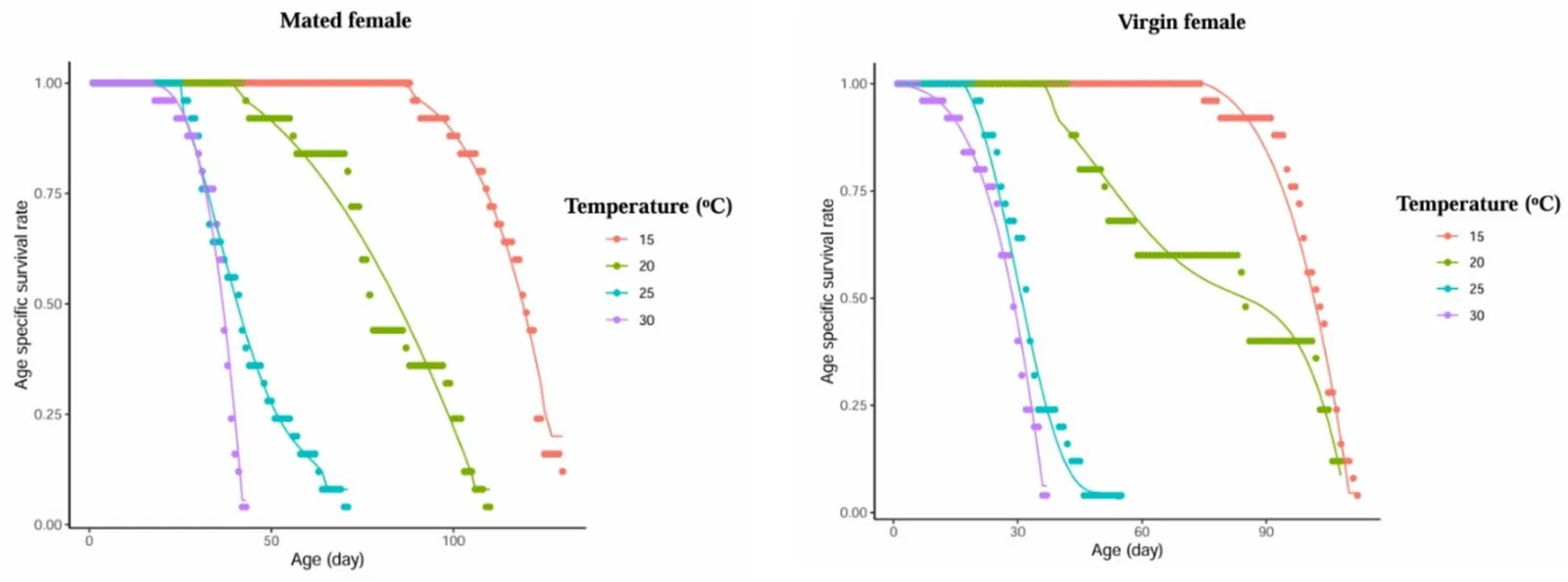
The age-specific survival (observed data: *solid dots* and Weibull distribution: *solid line*) of *Blattisocius mali* mated and virgin females at four different temperatures.

Daily age-specific survival rate, obtained by the Weibull function, of both mated and virgin *B. mali* females at different temperatures is illustrated in Fig. 2. Notably, the survival rate of *B. mali* females was higher at 20 °C and 25 °C than at other tested temperatures. In addition, it remained high for a relatively long period and declined sharply at all tested temperatures. The Weibull function fitted well to the survival data at all temperatures examined. Additionally, the *LT*_*50*_ values predicted by the Weibull function (*LT*_*50−P*_) are closely aligned to those obtained from the experimental data (*LT*_*50−E*_) (Table 5). The estimated values of the shape parameter *c* were greater than 1 at all tested temperatures, classifying the survival curve of *B. mali* into type I according to Deevey^42^ (Table 5, Fig. 2).

The estimated life table parameters of *B. mali* at four different temperatures are presented in Table 6. The intrinsic rate of increase (*r*) and finite rate of increase (λ*)* were similar at 30 °C and 25 °C and then decreased with decreasing temperature up to 15 °C. The gross reproductive rate (*GRR*) and net reproductive rate (*R*_*o*_) were similar at 20 °C and 25 °C but decreased with either increasing or decreasing temperature. The mean generation time (*T*) and doubling time (*DT*) were the longest at 15 °C and decreased with increasing temperature.

**Fig. 3:**
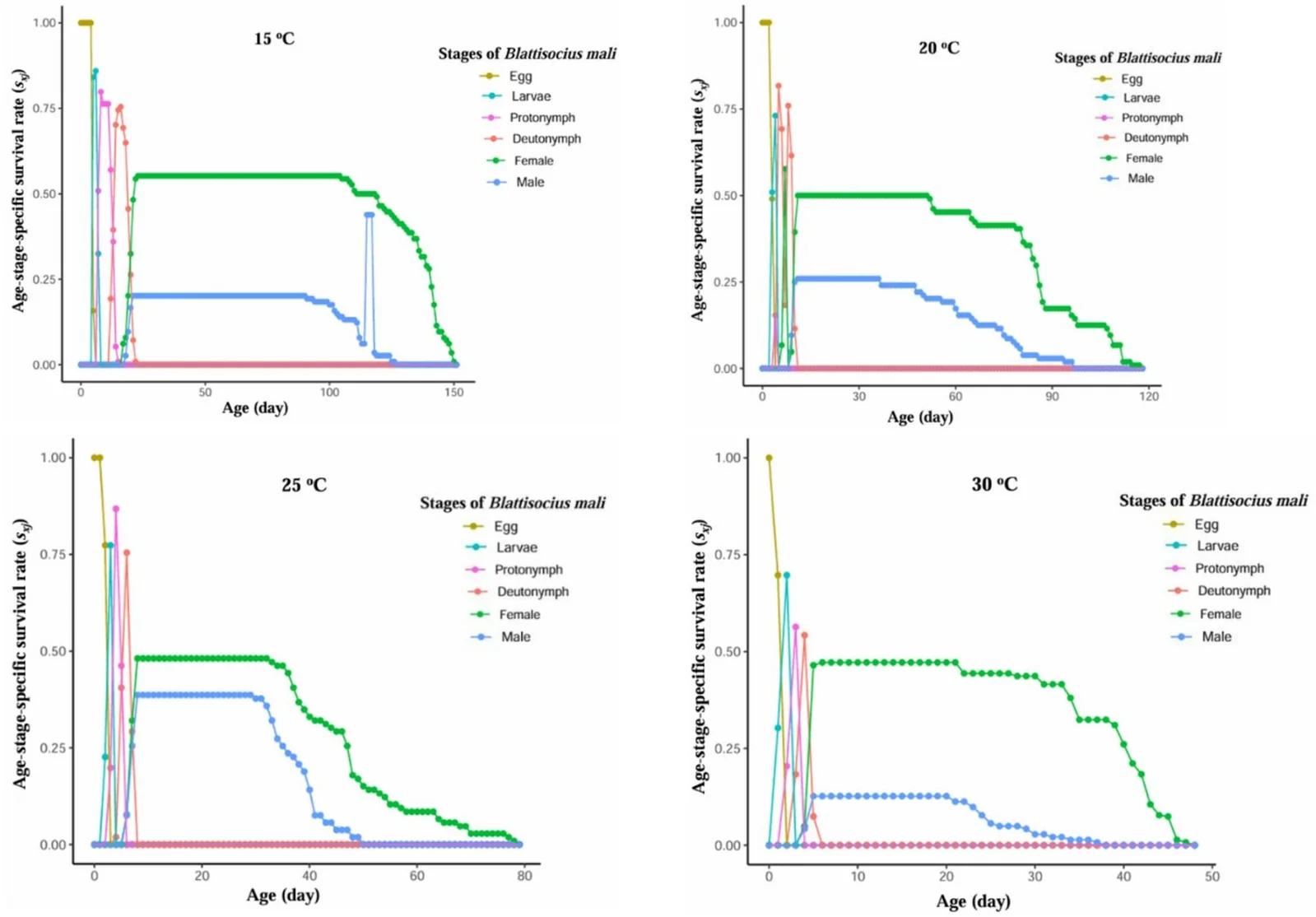
The age-stage-specific survival rate (*s*_*xj*_) of each life stage of *Blattisocius mali* at four different temperatures.

**Fig. 4:**
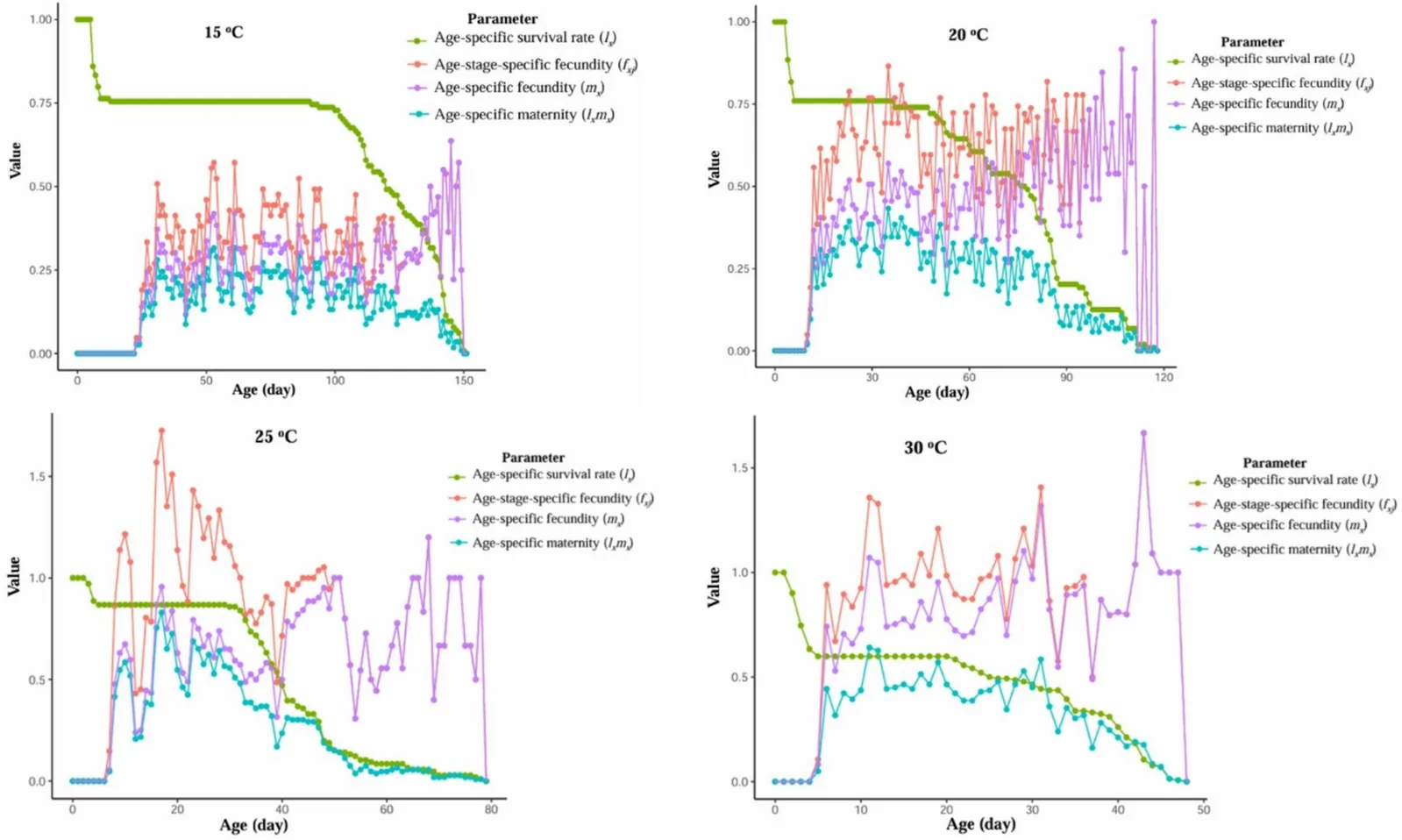
The age-specific survival rate (*l*_*x*_), age-stage-specific fecundity of the female stage (*f*_*xj*_), age-specific fecundity (*m*_*x*_), and age-specific maternity (*l*_*x*_*m*_*x*_) of *Blattisocius mali* at four different temperatures.

Age-stage-specific survival rate (*s*_*xj*_) of *B. mali* at different temperatures is presented in Fig. 3, which shows that there was significant overlap between curves at each temperature. This overlap happened due to the variation in the development times of different life stages of *B. mali*. Notably, the age-specific survival rate of *B. mali* dropped more slowly at 15 °C as compared to other tested temperatures (Fig. 4). The peak value of age-stage-specific fecundity (*f*_*xj*_) was recorded on day 17 at 25 °C, which was higher than that at other tested temperatures (Fig. 4). The curve of the age-specific fecundity (*m*_*x*_) revealed that egg-laying of *B. mali* started on day 5 and maximum egg-laying (1.67 eggs per day) of *B. mali* was recorded on day 45 at 30 °C, while the egg-laying of *B. mali* started on day 7 and maximum egg-laying (1.18 eggs per day) was recorded on day 17 at 25 °C. The egg-laying of *B. mali* started on day 10 and the maximum egg-laying (1 egg per day) was recorded on day 117 at 20 °C, while the egg-laying of *B. mali* started on day 23 and the maximum egg-laying (0.64 eggs per day) was recorded on day 145 at 20 °C (Fig. 4). The greatest age-specific maternity (*l*_*x*_*m*_*x*_) of *B. mali* (0.93 offspring) was observed at 25 °C on day 17, followed by 0.64, 0.43, and 0.32 offspring at 30 °C, 20 °C, and 15 °C temperatures on days 11, 35, and 53, respectively (Fig. 4).

**Table 1:** Generalized Linear Model (GLM) and ANOVA statistics for biological parameters of *Blattisocius mali* for main effects (temperature and type of individual) and their interactions.

Biological parameter	Temperature			Type of individual			Temperature × Type of Individual		
	df	*F*	*P*	df	*F*	*P*	df	*F*	*P*
Egg period	3	847.17	< 0.0001	1	0.25	0.6190	3	1.21	0.3030
Larval period	3	338.01	< 0.0001	1	34.21	< 0.0001	3	14.64	< 0.0001
Protonymphal period	3	1641.25	< 0.0001	1	1.15	0.2850	3	0.38	0.7700
Deutonymphal period	3	1591.90	< 0.0001	1	3.23	0.0732	3	0.24	0.8673
Adult pre-ovipositional period (APOP)	3	237.04	< 0.0001	1	5.07	0.0254	3	1.82	0.1444
Total pre-ovipositional period (TPOP)	3	2033.00	< 0.0001	1	2.80	0.0959	3	1.23	0.3000
Ovipositional period	3	296.38	< 0.0001	1	29.76	< 0.0001	3	1.86	0.1380
Life cycle	3	5807.20	< 0.0001	1	17.99	< 0.0001	3	2.09	0.1020
Fecundity	3	38.31	< 0.0001	1	123.01	< 0.0001	3	5.08	0.0021
Egg hatching percentage	2	91.59	< 0.0001	1	49.91	< 0.0001	2	14.82	< 0.0001
Adult longevity	3	486.72	< 0.0001	2	48.13	< 0.0001	6	3.76	0.0012

**Table 2:** Biological parameters (*Mean ± SE*) of *Blattisocius mali* at four different temperatures.

Biological parameter	Type of individual	Temperature (^o^C)			
		30	25	20	15
Egg period (day)	Female	1.8 ± 0.1Ad (*n* = 52)	2.7 ± 0.1Ac (*n* = 61)	3.4 ± 0.1Ab (*n* = 55)	5.2 ± 0.1Aa (*n* = 52)
	Male	1.7 ± 0.1Ad (*n* = 30)	2.8 ± 0.1Ac (*n* = 27)	3.5 ± 0.1Ab (*n* = 29)	5.1 ± 0.1Aa (*n* = 27)
Larval period (day)	Female	1.0 ± 0.0Ac (*n* = 52)	1.0 ± 0.0Ac (*n* = 61)	1.5 ± 0.0Ab (*n* = 55)	2.2 ± 0.0Aa (*n* = 52)
	Male	1.0 ± 0.0Ab (*n* = 30)	1.0 ± 0.0Ab (*n* = 27)	1.0 ± 0.0Bb (*n* = 29)	2.0 ± 0.0Ba (*n* = 27)
Protonymphal period (day)	Female	1.0 ± 0.1Ad (*n* = 52)	1.8 ± 0.1Ac (*n* = 61)	2.4 ± 0.1Ab (*n* = 55)	5.9 ± 0.1Aa (*n* = 52)
	Male	1.0 ± 0.1Ad (*n* = 30)	1.7 ± 0.1Ac (*n* = 27)	2.2 ± 0.1Ab (*n* = 29)	5.9 ± 0.1Aa (*n* = 27)
Deutonymphal period (day)	Female	1.2 ± 0.1Ad (*n* = 52)	1.8 ± 0.1Ac (*n* = 61)	2.8 ± 0.1Ab (*n* = 55)	6.7 ± 0.1Aa (*n* = 52)
	Male	1.0 ± 0.1Bd (*n* = 30)	1.7 ± 0.1Ac (*n* = 27)	2.8 ± 0.1Ab (*n* = 29)	6.5 ± 0.1Aa (*n* = 27)
Life cycle (day)	Female	4.9 ± 0.1Ad (*n* = 52)	7.3 ± 0.1Ac (*n* = 61)	10.1 ± 0.1Ab (*n* = 55)	20.1 ± 0.1Aa (*n* = 52)
	Male	4.7 ± 0.1Bd (*n* = 30)	7.2 ± 0.1Ac (*n* = 27)	9.5 ± 0.1Bb (*n* = 29)	19.5 ± 0.1Aa (*n* = 27)
Adult pre-ovipositional period (APOP) (day)	Mated female	1.2 ± 0.1Bc (*n* = 25)	1.0 ± 0.1Ac (*n* = 25)	1.1 ± 0.1Ab (*n* = 25)	6.6 ± 0.1Aa (*n* = 25)
	Virgin female	2.2 ± 0.3Ab (*n* = 25)	1.0 ± 0.3Ac (*n* = 25)	2.2 ± 0.3Ab (*n* = 25)	6.8 ± 0.3Aa (*n* = 25)
Total pre-ovipositional period (TPOP) (day)	Mated female	6.1 ± 0.2Ad (*n* = 25)	8.3 ± 0.2Ac (*n* = 25)	12.1 ± 0.2Ab (*n* = 25)	26.6 ± 0.2Aa (*n* = 25)
	Virgin female	7.1 ± 0.4Ac (*n* = 25)	8.3 ± 0.4Ac (*n* = 25)	12.2 ± 0.4Ab (*n* = 25)	26.9 ± 0.4Aa (*n* = 25)
Ovipositional period (day)	Mated female	33.5 ± 2.7Ac (*n* = 25)	41.8 ± 2.7Ac (*n* = 25)	79.4 ± 2.7Ab (*n* = 25)	109.1 ± 2.7Aa (*n* = 25)
	Virgin female	19.2 ± 3.0Bd (*n* = 25)	31.4 ± 3.0Bc (*n* = 25)	75.8 ± 3.0Ab (*n* = 25)	93.0 ± 3.0Ba (*n* = 25)
Post-ovipositional period (day)	Virgin female	5.2 ± 0.7 (*n* = 25)	–	–	–
Fecundity (eggs/female)	Mated female	33.7 ± 2.3Ac (*n* = 25)	42.1 ± 2.3Aab (*n* = 25)	48.3 ± 2.3Aa (*n* = 25)	39.2 ± 2.3Abc (*n* = 25)
	Virgin female	7.9 ± 1.9Bc (*n* = 25)	30.8 ± 1.9Ba (*n* = 25)	36.2 ± 1.9Ba (*n* = 25)	23.2 ± 1.9Bb (*n* = 25)
Egg hatching percentage (%)	Mated female	–	91.8 ± 1.2Aa (*n* = 25)	84.9 ± 1.2Ab (*n* = 25)	78.1 ± 1.2Ac (*n* = 25)
	Virgin female	–	87.9 ± 0.9Ba (*n* = 25)	80.3 ± 0.3Bb (*n* = 25)	58.6 ± 3.1Bc (*n* = 25)
Sex ratio (Female: Male)	Mated female	–	4.2 ± 0.7a (*n* = 25)	6.1 ± 0.7a (*n* = 25)	5.9 ± 0.7a (*n* = 25)
Adult longevity (day)	Mated female	34.7 ± 2.7Ac (*n* = 25)	42.8 ± 2.7Ac (*n* = 25)	81.3 ± 2.7Ab (*n* = 25)	115.7 ± 2.7Aa (*n* = 25)
	Virgin female	26.6 ± 3.0Bc (*n* = 25)	32.4 ± 3.0Bc (*n* = 25)	77.96 ± 3.02Ab (*n* = 25)	99.88 ± 3.02Ba (*n* = 25)
	Male	22.2 ± 2.2Cd (*n* = 25)	31.6 ± 2.2Bc (*n* = 25)	58.1 ± 2.2Bb (*n* = 25)	88.3 ± 2.2Ca (*n* = 25)

Uppercase and lowercase letters indicate significant differences (Tukey’s test; *P* < 0.05) between individuals and temperatures, respectively. *n indicates the number of replicates.

**Table 3:** Intercepts (*ώ ± SE*), slopes (*µ ± SE*), and *R*^*2*^*-values* estimated by linear regressions of developmental rates on temperature for different life stages of *Blattisocius mali* females and males fed on *Tyrophagus putrescentiae*, the estimated lower temperature threshold (*T*_*o*_) for development, and the development time expressed in thermal constant (k) (^*o*^*D ± SE*).

Life stages of *Blattisocius mali*	*ώ ± SE*	*µ ± SE*	*R*^2^-value	*T*_*o*_ (^o^C)	^*o*^*D* ± *SE*
Female					
Egg	− 0.285 ± 0.029	0.032 ± 0.001	0.789	8.91	34.674 ± 0.824
Larvae	− 0.120 ± 0.035	0.030 ± 0.002	0.807	4.00	22.943 ± 0.428
Protonymph	− 0.633 ± 0.016	0.050 ± 0.001	0.987	12.66	19.645 ± 0.456
Deutonymph	− 0.704 ± 0.024	0.052 ± 0.001	0.978	13.58	19.267 ± 0.443
Sum	− 0.563 ± 0.033	0.041 ± 0.0001	0.658	13.73	89.035 ± 3.633
Male					
Egg	− 0.291 ± 0.030	0.030 ± 0.001	0.777	9.70	34.862 ± 0.855
Larvae	− 0.114 ± 0.033	0.033 ± 0.002	0.794	3.45	22.678 ± 0.416
Protonymph	− 0.655 ± 0.016	0.058 ± 0.001	0.986	11.29	19.709 ± 0.417
Deutonymph	− 0.678 ± 0.026	0.056 ± 0.001	0.981	12.11	19.277 ± 0.438
Sum	− 0.573 ± 0.043	0.044 ± 0.001	0.4781	13.02	83.362 ± 3.633

**Table 4:** Maxima function parameter (*estimates ± SE*) for age-specific fecundity rate curves of adult females of *Blattisocius mali* at four different temperatures.

Parameter	Type of individual (*n* = 25)	Temperature (°C)			
		30	25	20	15
*α*	Mated female	0.180 ± 0.019	0.161 ± 0.019	0.046 ± 0.004	0.021 ± 0.003
	Virgin female	0.163 ± 0.019	0.158 ± 0.020	0.556 ± 0.004	0.028 ± 0.002
*β*	Mated female	0.056 ± 0.004	0.048 ± 0.004	0.023 ± 0.001	0.018 ± 0.002
	Virgin female	0.050 ± 0.004	0.042 ± 0.003	0.024 ± 0.001	0.028 ± 0.003
*F*_*max−E*_ (Age)	Mated female	1.50 (39)	2.12 (10)	1.00 (109)	0.87 (117)
	Virgin female	0.80 (2)	1.92 (11)	1.00 (102)	1.00 (112)
*F*_*max−P*_ (Age)	Mated female	1.37 (24)	1.83 (11)	0.93 (89)	0.82 (87)
	Virgin female	0.68 (21)	1.76 (10)	0.92 (76)	0.94 (83)
*R* ^*2*^	Mated female	0.974	0.976	0.972	0.982
	Virgin female	0.972	0.976	0.976	0.985

*n indicates the number of replicates. *F*_*max−E*_ and *F*_*max−P*_ are the maximum age-specific fecundity rates (eggs/female/day) obtained from the experimental data and predicted by the Maxima function, respectively. Numbers in parentheses indicate adult females’ age (day) when *F*_*max*_ occurs.

**Table 5:** Weibull function parameter (*estimates ± SE*) for age-specific survival curves of mated and virgin adult females of *Blattisocius mali* at four different temperatures.

Parameter	Type of individual (*n* = 25)	Temperature (°C)			
		30	25	20	15
*b*	Mated female	0.905 ± 0.298	0.675 ± 0.020	0.833 ± 0.027	0.975 ± 0.030
	Virgin female	0.876 ± 0.024	0.647 ± 0.021	0.878 ± 0.023	0.932 ± 0.024
*c*	Mated female	3.004 ± 0.280	1.672 ± 0.189	2.562 ± 0.267	4.520 ± 0.350
	Virgin female	3.362 ± 0.275	1.796 ± 0.156	2.678 ± 0.286	4.474 ± 0.353
*LT*_*50−E*_	Mated female	36	41	77	119
	Virgin female	28	32	84	102
*LT*_*50−P*_	Mated female	38	41	75	115
	Virgin female	27	30	82	97
*R* ^*2*^	Mated female	0.918	0.903	0.874	0.894
	Virgin female	0.906	0.928	0.868	0.884

*n indicates the number of replicates. *LT*_*50−E*_ and *LT*_*50−P*_ values, the mean time intervals during which 50% of the population may be expected to die, were obtained from the experimental data and predicted by the Weibull function, respectively.

**Table 6:** Life table parameters (*estimates ± SE*) of *Blattisocius mali* at four different temperatures. Different letters indicate significant differences (Bootstrap resampling technique [100000]; *P* < 0.05) between temperatures.

Life table parameters	Temperature (°C)			
	30	25	20	15
Intrinsic rate of increase (*r*) (day^− 1^)	0.14 ± 0.00**a**	0.16 ± 0.01**a**	0.10 ± 0.00**b**	0.05 ± 0.00**c**
Finite rate of increase (λ) (day^− 1^)	1.15 ± 0.01**a**	1.17 ± 0.01**a**	1.10 ± 0.00**b**	1.15 ± 0.00**c**
Gross reproductive rate (*GRR*) (offspring/individual)	36.5 ± 2.4**b**	52.4 ± 3.4**a**	50.6 ± 3.2**a**	36.2 ± 2.4**c**
Net reproductive rate (*R*_*o*_) (offspring/individual)	12.3 ± 1.4**c**	24.2 ± 2.2**a**	25.5 ± 2.6**a**	17.9 ± 1.9**b**
Mean generation time (*T*) (day)	17.6 ± 0.3**d**	20.4 ± 0.4**c**	33.8 ± 0.6**b**	60.5 ± 0.9**a**
Doubling time (*DT*) (day)	4.9 ± 0.5**c**	4.4 ± 0.5**c**	7.2 ± 0.6**b**	14.6 ± 1.0**a**

## Discussion

The results of the present study revealed that *B. mali* could successfully develop and reproduce at all tested temperatures. Temperature significantly affected all the biological and life table parameters of *B. mali* except the sex ratio. The developmental period of *B. mali* was shortened with rising temperature. The life cycle of females was significantly longer than that of males at 20 °C and 30 °C, while there was no significant difference at 15 °C and 25 °C. The lower temperature threshold and thermal constants ranged between 13.02 °C and 13.73 °C and 83.36 ^o^D to 89.04 ^o^D, respectively. The APOP, TPOP, and ovipositional period of both mated and virgin females shortened with rising temperature. Additionally, the fecundity of both mated and virgin females was higher at 20 °C and 25 °C than at 15 °C and 30 °C. Mated females had a longer ovipositional period and longevity, as well as higher fecundity and egg-hatching percentage, than virgin females at most of the tested temperatures. Age-specific fecundity and survival rates of both mated and virgin females were satisfactorily described by the Maxima and Weibull functions, respectively. The maximum daily fecundity rates of both mated and virgin females were obtained by the Maxima function at 25 °C. Based on Weibull functions, *B. mali* showed a Type I survivorship curve. The sex ratio of mated females was female-biased, and it did not vary with temperature. *Blattisocius mali* had significantly higher values of finite rate of increase and intrinsic rate of increase at 25 °C and 30 °C. In comparison, it had higher values of net reproductive rate and gross reproductive rate at 20 °C and 25 °C. The mean generation time and doubling time were the longest at 15 °C.

The metabolic rate of arthropods increases with a temperature rise, which accelerates the ageing process^4,43^. Increased temperature also shortened the developmental duration of *B. mali*, resulting in the shortest egg, larval, protonymphal, and deutonymphal periods, and the life cycle of *B. mali* females and males at 30 °C compared to other tested temperatures. A similar trend in the developmental duration with temperature has been reported for other blattisociid mites, *B. tarsalis*^38^, *Lasioseius sugawarai* (Ehara) (Acari: Blattisociidae)^39^, *Lasioseius* sp.^40^, and *Lasioseius japonicus* (Ehara)^8^. The developmental periods and life cycle of females and males obtained in this study were longer than those reported by Abbas et al.^18^, Pirayeshfar et al.^19,20^, and Asgari et al.^21^ at 25 °C, which might have resulted from different experimental set-ups and prey types used in these studies. For mite pests affecting stored agricultural products, employing a linear model provides a means to predict lower temperature thresholds and thermal constants, which are essential for devising temperature-modulation-based control strategies^44^. The lower temperature thresholds for the development of immature life stages of *B. mali* were estimated to be between 3.4 °C and 13.7 °C. The lower temperature thresholds for *T. putrescentiae* populations ranged between 4.4 °C and 10.5 °C^45^, which are closer to those values for *B. mali*. It suggests that *B. mali* can develop similarly to its prey *T. putrescentiae* at very low temperatures and control this acarid mite, and may be recommended for the management of acarid mite pests infesting stored products.

According to the general-purpose genotype hypothesis of Lynch^46^, animals exhibiting arrhenotokous development accumulate a broad array of adaptations that allow them to colonize and survive in many habitats, including marginal habitats. Arrhenotoky is a phenomenon in which males arise from unfertilized eggs and females arise from fertilized eggs^47^. This phenomenon has been reported in some insects^48^ and mites^49–51^. Unlike other blattisociid mites^47,52,53^, our present study confirmed that *B. mali* is arrhenotokous, i.e., *B. mali* has diploid females and haploid males. At 30 °C, the APOP in mated females was significantly shorter than that of virgin females, which might be due to the activation of the female reproductive mechanism caused by mating, prompting earlier oviposition. Moreover, the mated females of *B. mali* deposited significantly more eggs throughout their life spans than virgin females at all tested temperatures. This increase in egg production might be attributed to the resources mobilized through mating, enabling females to generate additional eggs. Unsurprisingly, the ovipositional period and longevity increased with mating; more eggs require a longer time interval to oviposit. The hatching success of *B. mali* eggs may be improved by mating, leading to a higher egg-hatching percentage in mated females. The sex ratio of the predatory soil mite *Hypoaspis miles* (Berlese) (Acarina: Hypoaspidae) was female-biased at all 4 temperatures, 15 °C, 20 °C, 25 °C, and 30 °C, and increased with temperatures up to 25 °C, decreasing at 30 °C^6^. In contrast, the present study showed that the offspring sex ratio of *B. mali* was female-biased at all tested temperatures but was not significantly influenced by temperature. Since fertilized eggs develop into females, the similarity in sex ratio indicates that adult activity and mating may not be affected by temperature fluctuations, reflecting the sufficient adaptation or ability to adapt to temperature extremes.

The mean total number of eggs per *B. mali* female, whether virgin or mated, ranged from 2 eggs at 30 °C for virgin females to 70 eggs at 20°C for mated females. Previous studies showed that *B. mali* mated females averaged 23, 42, 12, 3.5 eggs/female^9^, 42 eggs/female^20^, 76, and 198 eggs/female^21^ while preying on *T. putrescentiae* at 25 °C. Our study showed that the maximum fecundity of *B. mali* (49 eggs/female) was obtained at 20 °C, and the maximum daily fecundity (2.12 eggs/female/day) was recorded at 25 °C and fitted well to those predicted by the quadratic regression model. According to a previous study by Sánchez-Ramos & Castañera^36^, the maximum fecundity of *T. putrescentiae* (555 eggs/female) was obtained at 20 °C, and the maximum daily fecundity (24 eggs/female/day) was recorded at 25 °C. This similarity in the effect of temperature on the fecundity suggests that the favourable temperature for the physiological processes involved in egg laying and an increase in mite populations^54^ was similar for both *B. mali* and *T. putrescentiae*.

The time required to reach the peak fecundity rate was the shortest at 25 °C for both mated and virgin females. This time was slightly longer for mated females than for virgin females at all tested temperatures, which might be due to the longer ovipositional period in mated females. The peak fecundity rate was predicted to be at 25 °C for both mated and virgin females. The peak fecundity of mated edaphic predatory mite *Lasioseius* sp. females was reported at 28°C^8,9^ and 24°C^40^, aligning closely with the present findings. The higher *R*^*2*^ values for both mated and virgin females at all tested temperatures showed that the Maxima function effectively modeled the average daily fecundity data, successfully predicting the day of peak fecundity for all tested temperatures.

Based on the ideal patterns for the survival curves of Deevey^42^ and their correspondence with the shape parameter (*c*) of the Weibull function, it was shown that the mortality rate for both mated and virgin females increased with age, resulting in type I survivorship curves. The greater *R*^*2*^ values for both mated and virgin females at all tested temperatures showed that the Weibull function fitted satisfactorily with the population survival data. Additionally, the mean time intervals during which 50% of the population may be expected to die were shortened with a rise in temperature for both mated and virgin females.

The understanding gained by life table analyses can provide a more rational basis for devising pest management programs by identifying vulnerable life stages, as well as enhancing our ecological insight into the factors that affect a species’ population dynamics. Rising temperatures up to 25 °C resulted in a shorter life cycle, APOP, ovipositional period, and adult longevity of *B. mali*, which was reflected by an increase in the intrinsic rate of increase and finite rate of increase as well as a decrease in mean generation time and doubling time. Interestingly, *B. mali* females exhibited a higher fecundity at 20 °C and 25 °C, resulting in higher values of net reproductive rate and gross reproductive rate at these temperatures. The intrinsic rate of increase and finite rate of increase found for *B. mali* at 25 °C were lower than the values obtained by Pirayeshfar et al. and Asgari et al.^19–21^. Conversely, the mean generation time and doubling time found for *B. mali* at 25 °C were longer than the values obtained by Pirayeshfar et al. and Asgari et al.^19–21^. However, the net reproductive rate and gross reproductive rate found for *B. mali* at 25 °C were higher than those reported by Pirayeshfar et al.^19,20^ but lower than those reported by Asgari et al.^21^. These differences might be associated with the differences in the humidity level, e.g., Pirayeshfar et al.^19,20^ and Asgari et al.^21^ performed experiments at 65 ± 5% and 75 ± 5% RH, respectively, which are different from the humidity level used in our study (85 ± 5% RH).

In conclusion, this study provides fundamental insights into the effect of temperature on the biological and life table parameters of *B. mali*. The findings indicate that the edaphic predatory mite *B. mali* can develop and reproduce at temperatures between 15 °C and 30 °C, and the prey *T. putrescentiae* have approximately similar temperature thresholds for development, positioning it as a viable option for the biological control of acarid mite pests within this temperature range. *Blattisocius mali* shows arrhenotokous development. The maximum fecundity and maximum daily fecundity rate were obtained at 20 °C and 25 °C, respectively. The mortality rates of both mated and virgin females increase with the increase in their age. The populations of *B. mali* can increase more rapidly at 20 °C and 25 °C compared to other temperatures. The values of biological and life table parameters estimated in laboratory settings may not necessarily reflect the actual values attained in storage facilities, greenhouses, and crop fields. In addition, other factors, such as the spatial dynamics of pests and predators, the predators’ searching efficiency, and the biological control strategy, are likely to influence the outcome of biological control. Moreover, monitoring degree days serves as an effective approach for forecasting the development of the *B. mali* population. In addition, these data may be useful for developing mathematical models describing the population dynamics of this mite species and predicting the relationship between biological and life table parameters of mites and environmental factors. Such models will be used as tools for the establishment of optimal biological and management strategies. Further studies are necessary to expand the thermal range to determine the upper temperature threshold for the predator *B. mali*.

## Methods

### Mite colonies

The primary colony of *T. putrescentiae* was taken from the mass rearing of the Department of Plant Protection, Warsaw University of Life Sciences, Warsaw, Poland. To obtain a sufficient quantity of eggs and larvae, the *T. putrescentiae* culture was reared using the same quantity of instant dry bakers’ yeast and wheat bran in glass Petri dishes, at 26 °C and 95 ± 5% RH, kept in darkness in a Sanyo Environmental Test Chamber (Panasonic MLR-350, Japan)^29,30^. The separation of *T. putrescentiae* eggs from other life stages was performed by using a 100 μm mesh screen^29,30^. Petri dishes were placed on top of water-saturated foam inside larger plastic containers, partially filled with water, and covered with a lid containing small pores for ventilation. Once the population of *T. putrescentiae* became abundant in a colony, mites would migrate to the top of the Petri dishes. At this point, new colonies were established by transferring motile mites from the top of Petri dishes of the existing colony to prevent overcrowding and reduce the risk of fungal contamination.

The stock colony of *B. mali* was taken from the mite population of the Department of Plant Protection of Warsaw University of Life Sciences. This colony was maintained on soaked foam platforms with wheat bran as a substrate, following the procedure outlined by Michalska et al.^26–28^. The colony was fed with a mixture of life stages of *T. putrescentiae* weekly and maintained in a climatic room of the Department of Plant Protection, at 21–23 °C, 85 ± 5% RH, and 16 L/8D photoperiod. The *B. mali* colony was always renewed one week before starting the experiment, and mixed life stages of *T. putrescentiae* were always provided as food to the colony before 24 h of selecting females for obtaining the same-aged egg cohort at each temperature.

### Experimental set-up

The experimental unit consisted of the Plexiglass cage described by Jena et al.^29,30^, in which a conical-shaped hole was drilled instead of a round hole. A chamber was made by attaching a piece of white filter paper to the lower surface of the hole using paraffin wax, and a suitable glass coverslip was placed on its upper surface using suitable clips after keeping the mites in the chamber to prevent escape. The cages were placed in a desiccator containing a saturated solution of KCl and distilled water on the bottom, ensuring relative humidities of 85%, 84%, 84%, and 82.6% at temperatures of 15 °C, 20 °C, 25 °C, and 30 °C, respectively, placed in a Panasonic-cooled incubator (MIR-154-PE, Japan).

To prepare the same-aged egg cohort for life table experiments, well-fed gravid adult females of *B. mali* were randomly selected from the colony and kept individually in the Plexiglas cage chamber with *T. putrescentiae* eggs and larvae as prey. The cages with the *B. mali* female and its prey were placed in a desiccator in an incubator set at four different temperatures, 15 °C, 20 °C, 25 °C, and 30 °C, and a 16 L/8D photoperiod for 24 h. After 24 h, *B. mali* females and eggs, if more than one egg was laid, were removed from the chamber of each cage, and a single *B. mali* egg was kept with *T. putrescentiae* eggs and larvae as food in each cage. There was always a sufficient amount of food in each cage during the development of the *B. mali* egg. Deposited extra food was removed daily and replaced with new food. Survival and developmental period of *B. mali* from egg to adult were recorded daily. The presence of exuviae was considered evidence of moulting. After adult emergence, 25 female and male predators were paired, and 25 females were kept unmated at the above-mentioned temperatures. They were provided with food and monitored daily to record the APOP, ovipositional, and postovipositional periods, longevity, and fecundity until their death. Males who died during the trial were replaced with those maintained at the same temperature. Egg hatching percentage of eggs laid by both mated and virgin females, as well as sex ratio of mated females, were determined simultaneously by taking their eggs in the above-mentioned experiment at the respective temperatures and placing them separately with food in cages. The egg hatching percentage of a female individual was determined by dividing the number of eggs hatched by the total number of eggs laid by that individual. The sex ratio of a female individual was estimated by dividing the number of female offspring by the number of male offspring born from that individual (F: M).

### Data analysis

The lower temperature threshold (*T*_*0*_) for *B. mali* immature life stages and the developmental time in day-degree (^*o*^*D*) time scale were computed using the formulas suggested by Roe et al.^41^. The daily age-specific fecundity rate (*F*_*(x)*_) (eggs/female/day) of both mated and virgin females was predicted by the Maxima function^55^. Also, the maximum fecundity rates obtained from the experimental data (*F*_*max−E*_) and those predicted by the Maxima function (*F*_*max−P*_) were determined. The age-specific survival (*S*_*(t)*_) of both mated and virgin females was predicted by the Weibull function^42,56^. In addition, the mean time interval during which 50% of the population may be expected to die, obtained from the experimental data (*LT*_*50−E*_), and those predicted by the Weibull function (*LT*_*50−P*_) were calculated.

The data on biological parameters of *B. mali* were analysed using TWOSEX-MS Chart, a computer program for the age-stage two-sex life table analysis^9^, to obtain life table parameters. The life table parameters were estimated using a large cohort of immature data and a much smaller cohort of adult data (mated females and males)^57^. According to the method described by Chi^9^, the raw data were used to calculate the age-stage specific survival rate (*s*_*xj*_, where *x* = age in day and *j* = stage; the first stage is egg, the second stage is a larva, the third stage is protonymph, the fourth stage is deutonymph, the fifth stage is mated female, and the sixth stage is mated male), age-stage-specific fecundity (*f*_*xj*_), age-specific survival rate (*l*_*x*_), age-specific fecundity (*m*_*x*_), and life table parameters, including the net reproductive rate (*R*_*o*_), the gross reproductive rate (*GRR*), the intrinsic rate of increase (*r*), the finite rate of natural increase (*λ*), mean generation time (*T*), and doubling time (*DT*) to construct the age-stage two-sex life table. In the age-stage two-sex life table, the parameters *l*_*x*_, *m*_*x*_, *R*_*o*,_ *GRR*,* r*,* λ*,* T*, and *DT* were calculated using formulas suggested by Chi^9^, Birch^58^, and Goodman^59^.

The data on the biological parameters were subjected to a Generalized Linear Model (GLM) with a Poisson probability distribution and log link function to estimate the means and standard errors. The post hoc comparisons were made using Tukey’s test in R (Version 3.4.0). All data on the life table parameters of *B. mali* were subjected to bootstrap resampling with 100,000 replications to estimate the standard errors. Differences in the life table parameters of *B. mali* reared at different temperatures were compared using the paired bootstrap test^60^.

## Data Availability

The data used in this study are available upon request by email to the corresponding author, MKJ (manoj_jena@sggw.edu.pl).

## Declarations

### Research involving human and animal participants

This research did not involve any studies with human participants, other vertebrates, or protected or endangered species.
